# Dietary calcium phosphate strongly impacts gut microbiome changes elicited by inulin and galacto-oligosaccharides consumption

**DOI:** 10.1186/s40168-021-01148-0

**Published:** 2021-11-04

**Authors:** Jori Fuhren, Markus Schwalbe, Jos Boekhorst, Christiane Rösch, Henk A. Schols, Michiel Kleerebezem

**Affiliations:** 1 Host Microbe Interactomics Group, Wageningen University & Research, De Elst 1, 6708 WD Wageningen, The Netherlands; 2 Laboratory of Food Chemistry, Wageningen University & Research, Bornse Weilanden 9, 6708 WG Wageningen, The Netherlands

**Keywords:** Intestinal microbiota, Short chain fatty acids, Diet, Prebiotics, Calcium phosphate

## Abstract

**Background:**

Fructo-oligosaccharides (FOS), inulin, and galacto-oligosaccharides (GOS) are widely recognized prebiotics that profoundly affect the intestinal microbiota, including stimulation of bifidobacteria and lactobacilli, and are reported to elicit several health benefits. The combination of dietary FOS and inulin with calcium phosphate was reported to stimulate commensal *Lactobacillus* populations and protect the host against pathogenic Enterobacteriaceae, but little is known about the effects of GOS in diets with a different level of calcium phosphate.

**Methods:**

We investigated the microbiome changes elicited by dietary supplementation with GOS or inulin using diets with high (100 mmol/kg) and low (30 mmol/kg) calcium phosphate levels in adult Wistar rats. Rats were acclimatized to the respective experimental diets for 14 days, after which fecal material was collected, DNA was extracted from fecal material, and the V3‑V4 region of the bacterial 16S rRNA gene was amplified with PCR, followed by microbial composition analysis. In tandem, the organic acid profiles of the fecal material were analyzed.

**Results:**

Feeding rats non-supplemented (no prebiotic-added) diets revealed that diets rich in calcium phosphate favored members of the Firmicutes and increased fecal lactic, succinic, acetic, propionic, and butyric acid levels. In contrast, relatively low dietary calcium phosphate levels promoted the abundance of mucin degrading genera like *Akkermansia* and *Bacteroides*, and resulted in increased fecal propionic acid levels and modest increases in lactic and butyric acid levels. Irrespective of the calcium phosphate levels, supplementation with GOS or inulin strongly stimulated *Bifidobacterium*, while only high calcium phosphate diets increased the endogenous *Faecalibaculum* populations.

**Conclusions:**

Despite the prebiotic’s substantial difference in chemical structure, sugar composition, oligomer size, and the microbial degradation pathway involved in their utilization, inulin and GOS modulated the gut microbiota very similarly, in a manner that strongly depended on the dietary calcium phosphate level. Therefore, our study implies that the collection of detailed diet information including micronutrient balance is necessary to correctly assess diet-driven microbiota analysis.

Video Abstract

**Supplementary Information:**

The online version contains supplementary material available at 10.1186/s40168-021-01148-0.

## Introduction

More than 2 decades ago, prebiotics were defined as: “a non-digestible food ingredients that beneficially affect the host by selectively stimulating the growth and/or activity of one or a limited number of bacteria in the colon, and thus improves health” [1]. Over the years, this definition has gone through several changes, to reach its recent, broader, and commonly accepted version in 2015: “a substrate that is selectively utilized by host micro-organisms conferring a health benefit.” Notably, this definition does no longer restrict prebiotics to food ingredients or intestinal applications, and provides no indication or direction for the health benefit, leaving a lot of freedom in the use of the term prebiotic [2]. Nevertheless, only a few compounds are considered established prebiotics [2, 3]. At present, the most commonly accepted prebiotic substrates are galacto-oligosaccharides (GOS) and the fructose-containing polymers fructo-oligosaccharides (FOS) and inulin. Inulin and FOS differ substantially in chemical composition, structure, and production methods from GOS [4–6] and therefore could potentially stimulate different microbiota members depending on the species- or strain-specific carbohydrate utilization repertoire [7, 8]. Inulin is composed of a series of linear β-2,1-linked fructose molecules with a terminal α-1,2-linked glucose moiety and occurs naturally in a wide variety of vegetables and has a varying degree of polymerization (DP), ranging from 2 to 60. FOS is derived from inulin through chemical or enzymatical conversions to result in a mixture of β-2,1-linked fructose molecules with either a terminal glucose or fructose moiety resulting in a substrate consisting of a substantially lower DP (DP2-10) [4]. Lactose-based GOS is industrially produced by various microbial β-galactosidases that when using high lactose concentrations produce a variety of galacto-oligosaccharides when using high lactose concentrations. The chemical complexity of these GOS preparations (i.e., linkage types, DP, degree of branching) depends on the reaction conditions and the β-galactosidase enzyme used, resulting in a variety of chemically diverse GOS preparations [5, 9]. GOS and inulin are renowned for their strong bifidogenic effect [10–16] and a more modest stimulation of intestinal lactobacilli [14, 15, 17–19]. These prebiotics, either on their own or in combination with probiotics (i.e., as a synbiotic) have been reported to contribute to various health benefits including improved immune function in elderly (i.e., GOS) [16, 18], reduced symptoms in inflammatory bowel syndrome [20], improved body-weight management or regulate satiety [21, 22], reduced symptoms in Crohn’s disease [23], and reduced mortality rate in newborn infants in rural India [24].

Intriguingly, dietary FOS has also been shown to increase pathogenic enterobacteria populations when supplemented in low-calcium phosphate diets [14, 25], and to increase the translocations and severity of infection of *Salmonella enteriditis* [26]. However, high calcium phosphate diets could counteract the FOS-induced stimulation of enterobacteria and colonic permeability [27], contributing to attenuation of infection and diarrhea symptoms in *Salmonella* and enterotoxigenic *Escherichia coli* (ETEC) challenged rats [14, 28, 29]. The modulation of the endogenous microbiota is evidently a primary quality of dietary prebiotics and dietary fiber, and is also known to be modulated by other macronutrients (e.g., dietary fat and protein intake) [3, 30–32]. However, modulation of the endogenous microbiota can also be influenced through micronutrients, including bio-available minerals such as calcium phosphate, magnesium, zinc and (heme-)iron, or vitamins [14, 28, 33–36]. It has been proposed that normally insoluble dietary calcium phosphate complexes can dissolve in the acidic environment of the GI-tract, resulting in increased buffering capacity in the intestinal lumen and contributing to the precipitation of cytotoxic surfactants like secondary bile acids [37–39]. Since Gram-positive bacteria have been suggested to be more susceptible to bile acids, the precipitation of bile acids by calcium phosphate supports various Gram-positive commensals like *Lactobacillus* and *Bifidobacterium* [40]. Additionally, diets high in calcium phosphate were also shown to protect against heme-induced (as proxy for red meat) colonic epithelial hyperproliferation that is associated with increased colon cancer risk [41, 42] and has been associated with intestinal mucin-degrading *Akkermansia* [35]. Consequently, the interplay of prebiotics with dietary calcium phosphate, as well as other macro- and micro- nutrients (e.g., heme), is an important decider in prebiotic effects on the intestinal microbiota and the associated beneficial versus detrimental health effects.

In this study, the modulatory effects of dietary inulin and GOS on the endogenous microbiota and fermentation metabolites in low and high calcium phosphate backgrounds were studied in adult male Wistar rats. Specific genera within the microbiota responded to the dietary modulations (i.e., prebiotic supplementation and calcium phosphate levels), revealing that GOS and inulin elicited highly comparable microbiota composition modulations that were strongly dependent on the calcium phosphate levels in the diet. Furthermore, supplementation with GOS and inulin resulted in dietary calcium phosphate-dependent changes in colonic organic acid profiles.

## Materials and methods

### Animal experiments and study design

Two independent animal experiments were performed, each using specific pathogen-free male Wistar rats (Harlen, Horst, The Netherlands) at 8 weeks of age, with an average body weight of 339 g ± 17. Upon arrival, rats were housed individually in standard rodent cages, in a temperature (22‑24 °C) and humidity (50‑60%) controlled environment with a 12-h light-dark cycle. During both trials, rats received food and water ad libitum, were weighed twice a week, and food intake was monitored daily. The rats (*n* = 8 per diet group) were fed powdered AIN-93-derived diets [43], with a high fat content (200 g fat/kg), to mimic the composition of a Western human diet and were acclimatized to housing conditions and the group specific diets for 14 days. The diets (Table 1) were supplemented with either 40 g/kg cellulose (Arbocel type B899 JRS, Zutphen, The Netherlands), 40 g/kg Orafti® GR Inulin (BENEO-Orafti, Oreye, Belgium), or 40 g/kg Vivinal GOS (Friesland Campina, Domo-branch, Amersfoort, the Netherlands) and contained either 30 or 100 mmol/kg (= 4.08 or 13.60 g/kg) CaHPO_4_ (calcium phosphate; CaP_i_) (Acros organics, Thermo Scientific). The experimental diets were designated as high CaP_i_ with cellulose (Hca), high CaP_i_ with GOS (HcaGOS), high CaP_i_ with inulin (HcaInu), low CaP_i_ with cellulose (Lca), low CaP_i_ with GOS (LcaGOS), and low CaP_i_ with inulin (LcaInu) (Figure SF1; Table ST1). The Lca, Hca, and HcaInu groups were included in both independent experiments, effectively generating two independent (*n* = 8) replicates for the Lca, Hca, and HcaInu groups. The low-CaP_i_ diets reflect the lower range of habitual dietary calcium intake in many regions throughout Asia, Africa, and South America, while the high-CaP_i_ diets reflect the upper boundaries of habitual dietary calcium intake in Northern European populations that consume increased amounts of dairy products that are rich in CaP_i_ [27, 44, 45]. After the 14-day acclimatization period, fecal pellets were collected during the morning directly from individual rats. Fecal pellets were weighed (average of 100 mg) and homogenized after the addition of a fixed volume of PBS (1 or 2 mL) using a T10 basic ULTRA-TURRAX® (IKA-Werke, Staufen im Breisgau, Germany). Fecal slurries were aliquoted (aliquots of 250 or 500 μl, respectively) and stored at −80 °C for later analyses (see below).

**Table 1:** Composition of the experimental diets^a^

Diet (g/kg)	Hca	HcaGOS	HcaInu	Lca	LcaGOS	LcaInu
Acid casein	200	200	200	200	200	200
Corn starch	241	232	239	245	237	243
Dextrose	241	232	239	245	237	243
Palm oil	160	160	160	160	160	160
Corn oil	40	40	40	40	40	40
Cellulose	60	20	20	60	20	20
Inulin	0	0	44^d^	0	0	44^c^
GOS	0	57.14^c^	0	0	57.14^c^	0
Vitamin mix^b^	10	10	10	10	10	10
Mineral mix^b^	35	35	35	35	35	35
CaP_i_ (CaHPO_4_)	13.56	13.56	13.56	4.08	4.08	4.08
Total	1000	1000	1000	1000	1000	1000

^a^High CaP_i_ (Hca); high CaP_i_, GOS (HcaGOS); high CaP_i_, inulin (HcaInu); low CaP_i_ (Lca); low CaP_i_, GOS (LcaGOS); low CaP_i_, inulin (LcaInu)

^b^The composition of the vitamin and mineral mixtures is according to the recommendation of the American Institute of Nutrition 1993 [43], except that calcium was omitted. In addition, tripotassium citrate was added instead of KH_2_PO_4_ and choline chloride was added instead of choline tartrate

^c^To acquire 40 g GOS/kg, the amount of GOS was increased since Vivinal GOS contains approximately 30% glucose, galactose, and lactose

^d^To acquire 40 g inulin/kg, the amount of inulin was increased since Orafti GR® inulin contains approximately 10 % glucose, fructose, and sucrose

### Fecal DNA extraction

DNA was extracted from the fecal aliquots using the repeated bead beating method [46] and the QIAamp Fast DNA Stool Mini Kit (Qiagen, Hilden, Germany), according to the manufacturer’s instruction. An average of 25 mg (standard deviation of 13 mg) of fecal sample (wet weight) was used as starting material for DNA extraction. Cells were lysed in Lysing Matrix B tubes prefilled with 0.1 mm silica beads (MP Biomedicals, Santa Ana, California, USA) using FastPrep-24TM (MP Biomedicals) at 5.5 m/s for 3 min (with intermittent cooling on ice in between after every minute). Subsequently, samples were heated at 95 °C for 15 min, supernatants were collected after centrifugation (10 min, 16 000 × *g*, 4 °C) and DNA was precipitated with 2.5 M ammonium acetate, followed by addition of one volume of isopropanol and incubated on ice for 30 min. Following precipitation, RNA and protein were removed from the samples by DNase-free RNase (10 mg/ml, Qiagen) and Proteinase K (Qiagen) treatment. Subsequently, DNA was purified using the QIAamp DNA Stool Mini Kit (Qiagen) as previously described [46]. DNA quality and quantity were determined using a Nanodrop DeNovix DS-11 spectrophotometer (DeNovix Inc., Wilmington, DE USA) and Qubit® 4 fluorometer (Thermo Fisher Scientific, Waltham, Massachusetts, USA) with the QubitTM dsDNA BR assay kit (Thermo Fisher Scientific), respectively. Total DNA yield resulted in an average of 1388 mg within a range of 232 to 3416 ng (data not shown), and for all samples PCR-amplicons were successfully obtained using the V3 and V4 primers.

### Amplification and sequencing of 16s rRNA gene

The V3‑V4 region of the bacterial 16S rRNA gene was amplified with PCR using the 341F primer (5′ – CCTAYGGGRBGCASCAG – 3′) and the 806R primer (5′ – GGACTACNNGGGTATCTAAT – 3′) as specified by Novogene (https://en.novogene.com/services/research-services/metagenomics/16s-18s-its-amplicon-metagenomic-sequencing/). Purified amplicons were subjected to extension PCR using barcoded Illumina universal index sequencing adaptors prior to sequencing. Samples were sequenced (paired-end) using the Illumina MiSeq system (experiment 1) or Illumina Novoseq system (experiment 2) (performed by Novogene Co., Ltd., Beijing, China). Different sequencing techniques were used because in-house protocol changes at Novogene in between the execution of the two experiments. Paired-end reads were assigned to samples based on their unique barcode and truncated by cutting off the barcode and primer sequence.

### Microbiota analysis

The analysis of the microbiome followed a previously proposed work-flow using the DADA2 package (v.1.12.1) [47] in R (v.3.6.1). Quality control included the filtering of low-quality reads, followed by read-dereplication, and amplicon sequence variants (ASVs) were inferred individually for forward and reverse reads. Subsequently, read-pairs with overlap were merged, while concatenating read pairs with overlap smaller than 12 base pairs. Singletons of concatenated reads were removed from further analysis to reduce computational intensity. Chimera removal was carried out and taxonomy was assigned using a 16S SILVA reference database (v.132). All previous steps leveraged the DADA2 package in R. The 16S rRNA amplicons were prepared, sequenced, and analyzed separately for the two independent experiments, as the difference in sequencing platform (MiSeq vs Novoseq) requires slightly different approaches in data processing. ASV calling was done with DADA2 with a post hoc work around to account for PHRED score quality binning in NovoSeq data that interferes with error model learning in DADA2. For this, the initially learned error model matrix was manually modified to ensure monotonicity for the error profile by clipping it to a maximum value, determined by the error frequency at Q40. For experiment 1 (MiSeq library), 4,200,134 reads were collected with an average of 131,254 reads per sample, and 4149 ASVs were detected (857 with ≥ 2 counts). For experiment 2 (Novoseq library), 3,959,851 reads were collected with an average of 98,996 reads per sample, and 3474 ASVs were detected (600 with ≥ 2 counts). For downstream analysis of the microbiota composition changes in the two experiments (separate or in combination; see the “Results” section), the ASV table was aggregated at phylum, family, and genus level and transformed into compositional values using phyloseq (version 1.28.0) [48] and microbiome packages (version 1.6.0) [49], discarding ASVs with a prevalence below 10%. For multivariate analysis at ASV level, ASV’s present in only a single sample and/or with a total count lower than 10 were discarded. For alpha diversity, the ASV table was filtered to contain at least 2 reads, no prevalence or variance filter was applied, rarified, and trimmed mean of *M* values (TMM) transformation was applied and Shannon diversity and Chao1 richness were calculated with the internal software in Microbiome Analyst [50]. Bray-Curtis dissimilarity was calculated in CANOCO 5 (Microcomputer Power, Ithaca, NY, USA) [51]. Compositional bar plots were created using ggplot2 (v.3.2.1) [52]. All scatter and boxplots for diversity indices, relative microbiota abundance, and fecal acid metabolite concentrations were created using GraphPad Prism version 8.2.1 for Windows (GraphPad Software).

### Fecal organic acid quantification

For lactic and succinic acid determination by high performance liquid chromatography (HPLC), supernatants of the homogenized fecal aliquots (see sections above) were collected by centrifugation (10 min, 30 000 × *g*, room temperature). These fecal homogenate supernatants were injected (10 μL) using an Ultimate 3000 HPLC equipped with an autosampler, a RI-101 refractive index detector (Shodex, Kawasaki, Japan), and an ion-exclusion Aminex HPX-87 H column (7.8 mm × 300 mm) with a guard column (Bio-Rad, Hercules, CA, USA). Elution used 5 mM H_2_SO_4_ at a flow rate of 0.6 mL/min at an oven temperature of 65 °C. Calibration curves of lactic and succinic acid were prepared in a range of 0.01‑1 mg/mL. Chromeleon 7.2 software (Thermo Fischer Scientific) was used for data analysis.

The short chain fatty acids (SCFA), acetic, propionic, and butyric acid were quantified in aliquoted fecal samples (see sections above) by gas chromatography equipped with a flame ionization detector (GC-FID). Standard mixtures of acetic, propionic, and butyric acid in the concentration range of 0.01‑3 mg/mL were included in the analysis. Each sample was mixed in a 2:1 ratio with a solution containing HCl (0.3 M), oxalic acid (0.09 M) to fully protonate the organic acids, and the internal standard 2-ethyl butyric acid (0.45 mg/mL). The mixture was incubated for 30 min at room temperature and debris was removed by centrifugation (5 min, 15,000 × *g*, room temperature), and supernatants (0.3 μL) were injected in a CP-FFAP CB column (25 m × 0.53 mm × 1.00 μm) (Agilent Technologies, Santa Clara, CA, USA). The temperature profile during GC analysis was initiated with 30 s at 100 °C, followed by raising (8 °C/min) the temperature to 180 °C, which was maintained for 1 min followed by raising (20 °C/min) the temperature to 200 °C at 20 °C/min and maintaining this temperature for 5 min. Glass wool was inserted in the glass liner of the split injection port to protect the column from contamination [53]. The Xcalibur software (Thermo Fischer Scientific) was used for data analysis.

### Statistical analysis

Significant differences between all groups for alpha and beta diversity were determined using Kruskal-Wallis ANOVAs with post hoc Dunn’s tests (*α* = 0.05) using Graphpad Prism version 8.00 for Windows (Graphpad Software). Significant differences between microbiota abundance and fecal acid profiles in the different groups of rats (supplemented versus non-supplemented within discriminant CaP_i_ diet, and high- versus low-CaP_i_ diets with analogous supplementation) were detected by a two-sided non-parametric Mann-Whitney *U* tests (*α* = 0.05) using GraphPad Prism version 8.00 for Windows (Graphpad Software). Principal component analyses (PCA) and partial redundancy analyses (pRDA) were performed in CANOCO 5, according to the user manual [51] with log transformed (*Y*′ = Y*1+1000) relative abundance at genus or ASV level. Arrows in PCA and pRDA plots correspond to microbial genera that are predicted to be correlated to environmental (or supplementary) variables. The top 10 genera with the largest contribution to the environmental variables in the ordination space were displayed. Significance of the explained variation was calculated by Holms corrected *p* value with *α* = 0.05.

## Results

### Animal experiments and study design

Eight-week-old male Wistar (*n* = 8 per group) rats were acclimatized to standardized AIN-93-based diets, supplemented with inulin, GOS, or cellulose with high or low CaP_i_ for 2 weeks. Dietary groups included high CaP_i_ (Hca), high CaP_i_ with GOS (HcaGOS), high CaP_i_ with inulin (HcaInu), low CaP_i_ (Lca), low CaP_i_ with GOS (LcaGOS), and low CaP_i_ with inulin (LcaInu). The Hca, HcaInu, and Lca groups were involved in two independent experiments, resulting in two (*n* = 8) independent replicates (Figure SF1). Feed intake stabilized from an average consumption of approximately 19 g ± 5 to 17 g ± 1.5 a day during the 2-week acclimatization time. Average rat weight increased from 339 ± 17 to 381 ± 17 g with an average relative increase of bodyweight of 6.8% and 5.3% for the first and second week, respectively. Average weight between groups and relative weight increase throughout the trial did not differ significantly throughout the acclimatization period (Figure SF3).

### Global microbiota composition across different dietary regimes

**Fig. 1:**
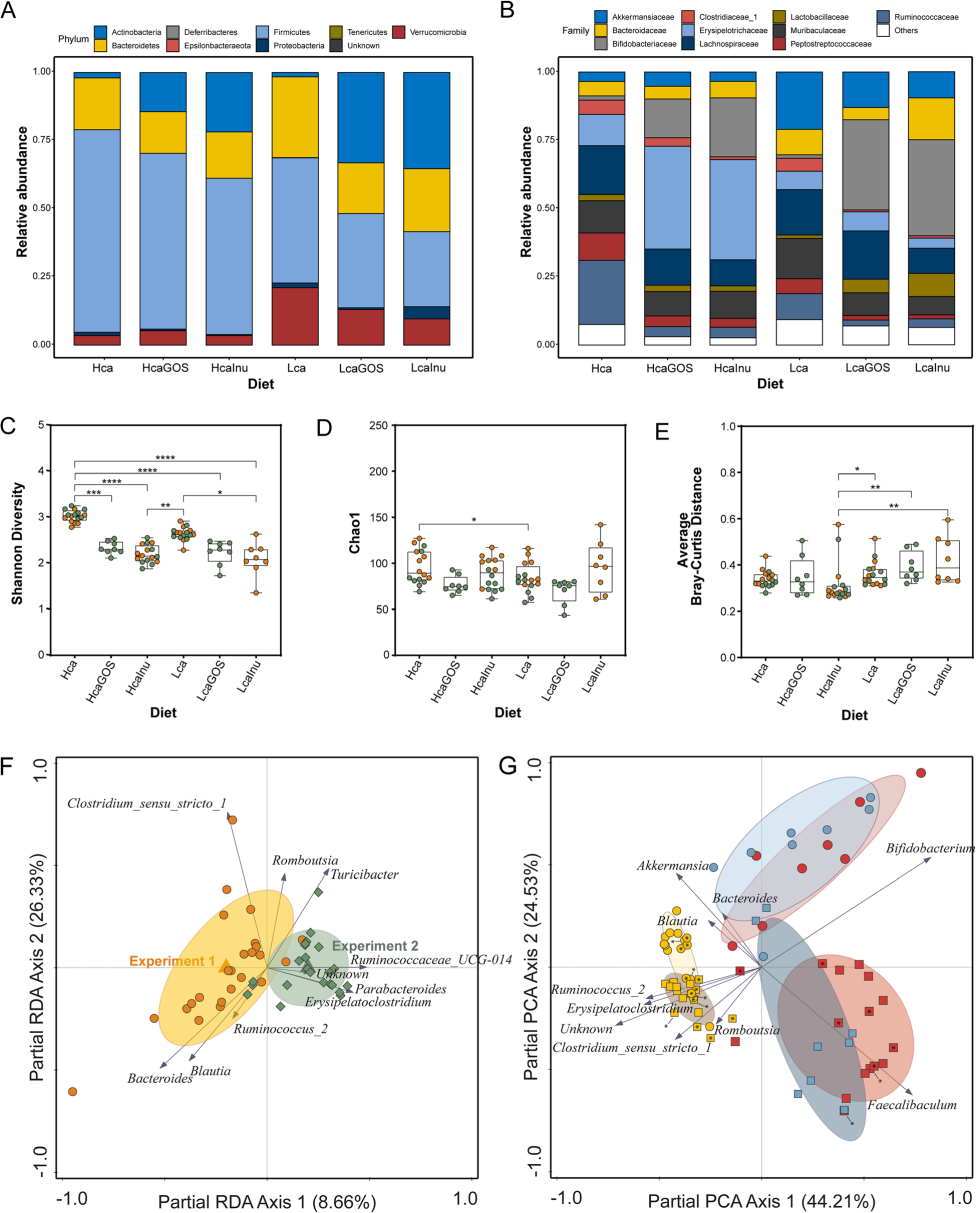
Diet-group specific average composition of microbiota in adult rats after 14 days of dietary acclimatization. Stacked bar plots of relative abundance of endogenous microbiota at **A** phylum level, and **B** family level. Alpha diversity with **C** Shannon diversity and **D** Chao1 richness as diversity indices. **E** Beta diversity with the average Bray-Curtis distance of each sample within each dietary group. Statistical significance between groups is tested with a Kruskal-Wallis ANOVA followed post-hoc Dunn’s multiple comparison tests. Asterisks (*) indicate significance between groups with certain *p* values. *p < 0.05; **p< 0.01; ***p < 0.001; ****p < 0.0001. Samples derived from experiment 1 and experiment 2 (Fig. 1F) are colored orange and green, respectively. **F** pRDA of the microbiota with experiment as explanatory variable (corrected for diet) showing the 10 genera with the strongest contribution to the discrimination between independent experiments. **G** Partial principal component analysis (PCA) (corrected for experiment) displaying the top 10 most discriminating genera, with low-CaP_i_ samples (circles), high-CaP_i_ samples (squares), GOS supplementation (blue), inulin supplementation (red), or un-supplemented (yellow). Sample labels for experiment 1 and experiment 2 (Fig. 1F) contain an asterisk (*) or are empty, respectively

Comparing the microbiota composition of rats on dietary regimes without prebiotic supplementation (i.e., Hca and Lca diet groups) revealed that the Hca diet group was associated with increased relative abundance of the Firmicutes phylum, whereas increased relative abundance levels of Bacteroidetes and Verrucomicrobia were found in the Lca group (Fig. 1A; Figure SF2.A). Dietary supplementation with GOS or inulin resulted in an obvious increase in Actinobacteria, consisting entirely of Bifidobacteriaceae at family level, in both the high- and low-CaP_i_ dietary background (Fig. 1A‑B; Figure SF2.A-B). In both CaP_i_ backgrounds, the increase in Actinobacteria coincided with a decrease in relative abundance of the Firmicutes, which in the low-CaP_i_ diets was accompanied by reduction of the relative abundances of Verrucomicrobia and Bacteroidetes. Particularly, within the Firmicutes phylum GOS or inulin supplementation resulted in distinct population shifts depending on the CaP_i_ level of the diet. In both dietary CaP_i_ regimes, GOS or inulin supplementation resulted in consistent decreases of Clostridiaceae_1, Peptostreptococcaceae and Ruminococcaceae, whereas Lachnospiraceae communities appeared to be much less affected. Notably, contrary to the decrease in relative abundance of the overall Firmicutes phylum, the high-CaP_i_ diet supplementation with GOS or inulin drastically increased the relative abundance of the Erysipelotrichaceae within this phylum, which was not seen in the low-CaP_i_ diets. The decrease in Verrucomicrobia and Bacteroidetes in the low-CaP_i_ diets after prebiotic supplementation (i.e., GOS or inulin) is reflected by the decrease in Akkermansiaceae, Bacteroidaceae, and Muribaculaceae.

The genus-level microbiome alpha diversity (Shannon Diversity index) was not significantly different in the gut of rats of the Lca group compared to those of the Hca group. However, GOS or inulin supplementation significantly reduced the diversity, which was especially apparent in Hca diet groups, but was also detected to a lesser extent in the Lca groups (Fig. 1C). Conversely, when also taking richness into account (Chao1 index), these differences were much less pronounced, although the diversity was still slightly lower in rats that were fed the Lca diet compared to rats on Hca diet (Fig. 1D). Beta-diversity was significantly increased for all low-CaP_i_ diets (i.e., Lca, LcaGOS, and LcaInu) compared to the HcaInu diet, but no differences were observed compared to the Hca and HcaGOS diets (Fig. 1E).

Because several dietary groups (i.e., Hca, Lca and HcaInu) are represented by groups of rats derived from independent experiments, we could assess the experimental variation using partial redundancy analysis (pRDA) corrected for diet and the “experiment” as explanatory variable (Fig. 1F). There is a significant link between experiment and microbiome composition (explained variation 8.7%, *p* = 0.0002). The difference between the independent experiments is largely driven by increased relative abundances of *Parabacteroides*, *Ruminococcaceae_UCG-014* and an unknown genus in the second experiment, while the first experiment was characterized by higher relative abundances of *Bacteroides* and *Blautia.* Therefore, to avoid the results are confounded by the variations driven by the independent experiments, the “experiment” (first or second) was consistently corrected for as a co-variate in the downstream multivariate analyses.

To identify the strongest differentiating genera per dietary regime, a partial principal component analysis (PCA) at genus level shows a clearly separate clustering of the GOS or inulin supplemented diets relative to the un-supplemented diets (Lca or Hca) (Fig. 1G). The analysis did not separate microbiota composition clusters on basis of the prebiotic supplement used, but the exact way in which they are jointly different depends on the CaP_i_ level of the diet. This result indicates that GOS and inulin supplementation elicit congruent effects on the microbiota composition in rats, but these effects are strongly dependent on the CaP_i_ level of the diet. The microbiota observed in rats that were fed the background diets without supplementation (i.e., Lca and Hca groups) appeared to be only partially separated on basis of the CaP_i_ level in this analysis, indicating that the microbiota impact of the dietary CaP_i_ level is substantially smaller than the effect of the prebiotic supplementation, which strongly enhanced the distinction between the Lca and Hca groups irrespective of the prebiotic used. Notably, a partial PCA using ASV-level composition data provided similar results compared to analysis at genus level, with highly congruent relative positioning of the different diet groups and very similar explained variations (45.67% on *X*-axis versus 24.96% on *Y*-axis; data not shown).

### Calcium phosphate is an important modulator of the endogenous microbiota

**Fig. 2:**
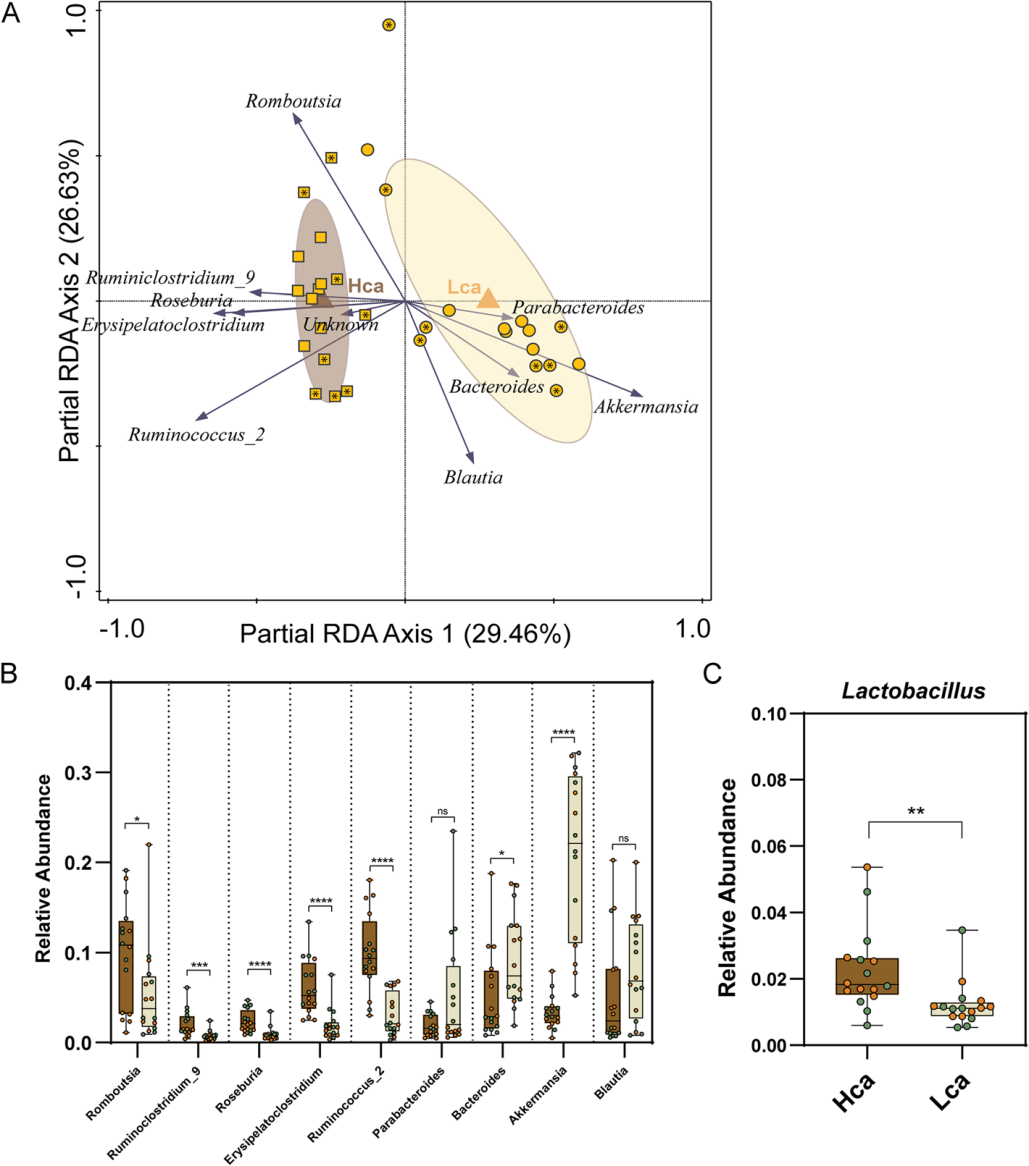
Effect of dietary calcium phosphate on the microbiota. **A** pRDA for the explanatory variable CaP_i_ (corrected for experiment) in the un-supplemented control diets Lca (circles) and Hca (squares), displaying the 10 most discriminative genera. The Hca versus Lca diet explains 29.46% of the microbiota variation (*p* value 0.002). Sample labels for experiment 1 and experiment 2 (Fig. 1F) contain an asterisk (*) or are empty, respectively. **B** Univariate analysis of the 9 named and most discriminative (ignoring the “unknown”) genera revealed by pRDA of the Hca (dark brown) and Lca (beige) diet groups. **C** Univariate analysis of *Lactobacillus* in Hca and Lca diet groups (*p* = 0.0012). Asterisks (*) indicate significance after a two-sided Mann-Whitney *t* test. *p < 0.05; **p < 001; ***p < 0.001; ****p < 0.0001; ns, not significant. Samples derived from experiment 1 and experiment 2 (Fig. 1F) are colored orange and green, respectively

**Fig. 3:**
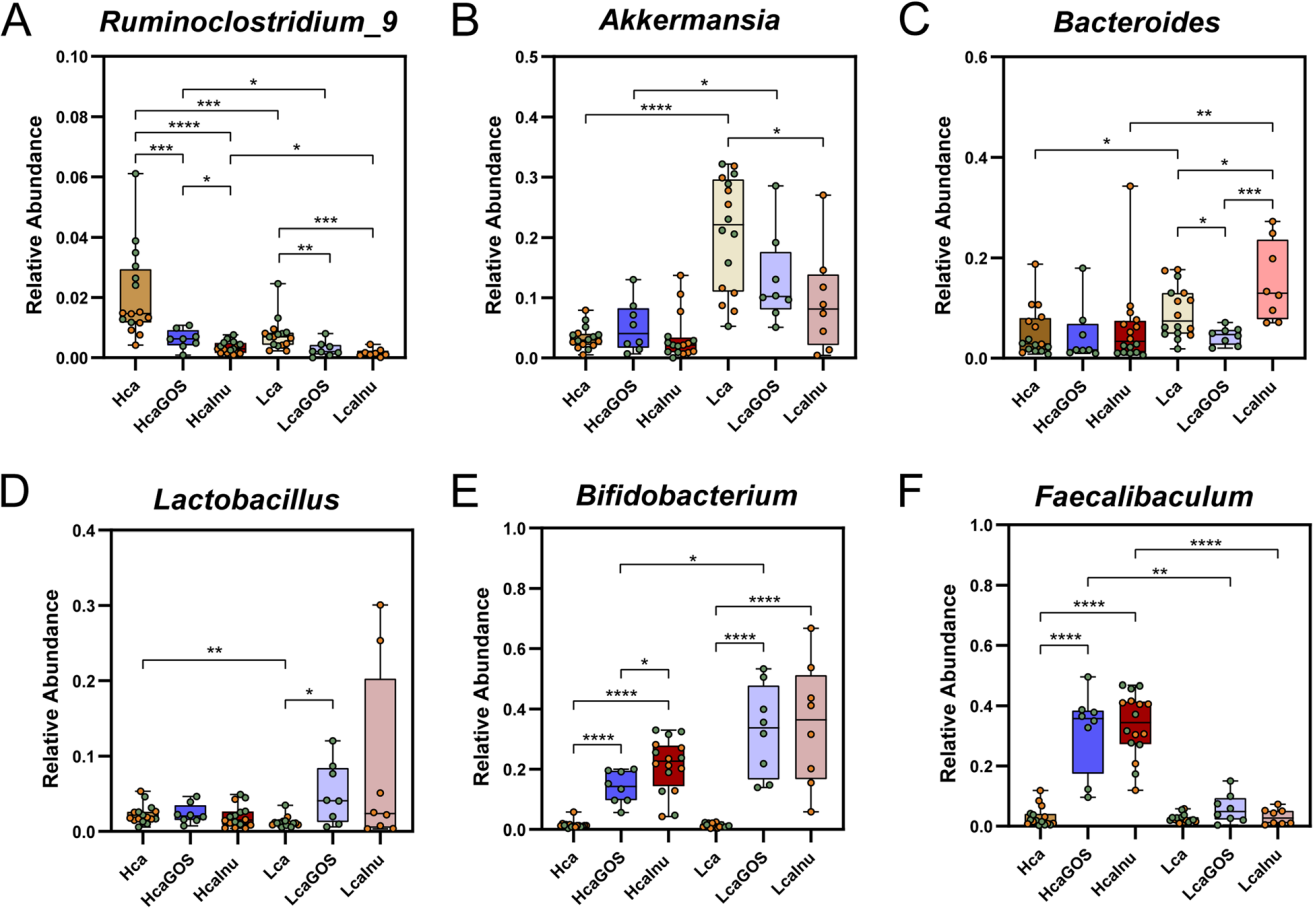
Univariate analysis of selected discriminant genera between dietary groups. Relative abundance of the genera **A** *Ruminoclostridium*, **B** *Akkermansia*, **C** Bacteroides, **D** *Lactobacillus*, **E** *Bifidobacterium*, and **F** *Faecalibaculum* in each dietary group. Asterisks (*) indicate significance after a two-sided Mann-Whitney *t* test. *p < 0.05; **p < 001; ***p < 0.001; ****p < 0.0001. Samples derived from experiment 1 and experiment 2 (Fig. 1F) are colored orange and green, respectively

Since the modulatory effects of GOS and inulin seem to greatly depend on the CaP_i_ levels in the diet, the primary differences in the high- and low-CaP_i_ control diets (i.e., Hca and Lca groups) were further investigated to identify CaP_i_-dependent microbiota modulations. pRDA shows a strong link between microbiome composition and CaP_i_ level (explained variation of 29.5%, *p* = 0.0002) (Fig. 2A). Increased relative abundance of the genera *Ruminiclostridium_9*, *Roseburia*, *Erysipelatoclostridium*, *Ruminococcus_2*, and *Romboutsia* is associated with the Hca diet group, whereas increased relative abundance of *Bacteroides* and *Akkermansia* is associated with the Lca diet group. Based on previously reported increased abundance of intestinal *Lactobacillus* populations in rats that were fed a high-CaP_i_ diet [14, 28], we specifically analyzed this genus confirming that rats fed the Hca diet was correlating with higher relative abundances of *Lactobacillus* although the average relative abundance of this genus was relatively low (2.3% and 1.2% for Hca and Lca groups, respectively) (*p* value of 0.0012) (Fig. 2C). The impacts of dietary CaP_i_ on the gut microbiota composition were largely conserved in rats that were fed the supplemented variants of these diets (i.e., LcaGOS and LcaInu versus HcaGOS and HcaInu). This is illustrated by the significant differences in *Ruminoclostridium_9*, *Akkermansia*, *Bacteroides* (Fig. 3A‑C), and the genera *Romboutsia*, *Roseburia*, and *Erysipelatoclostrdium* (Figure SF4) when comparing analogous Lca and Hca diets with the same supplementation, suggesting that the supplementation of GOS and inulin did not prominently alter CaP_i_-driven microbiota differences. In contrast, such conservation of CaP_i_ effects on the microbiota were neither detected for *Ruminococcus_2* (Figure SF4), nor for *Lactobacillus*, where the latter genus appeared to be particularly increased in several rats that were fed LcaGOS and LcaInu diets (Fig. 3D).

### GOS- or inulin-supplementation elicit congruent, calcium phosphate-dependent microbiota changes

**Fig. 4:**
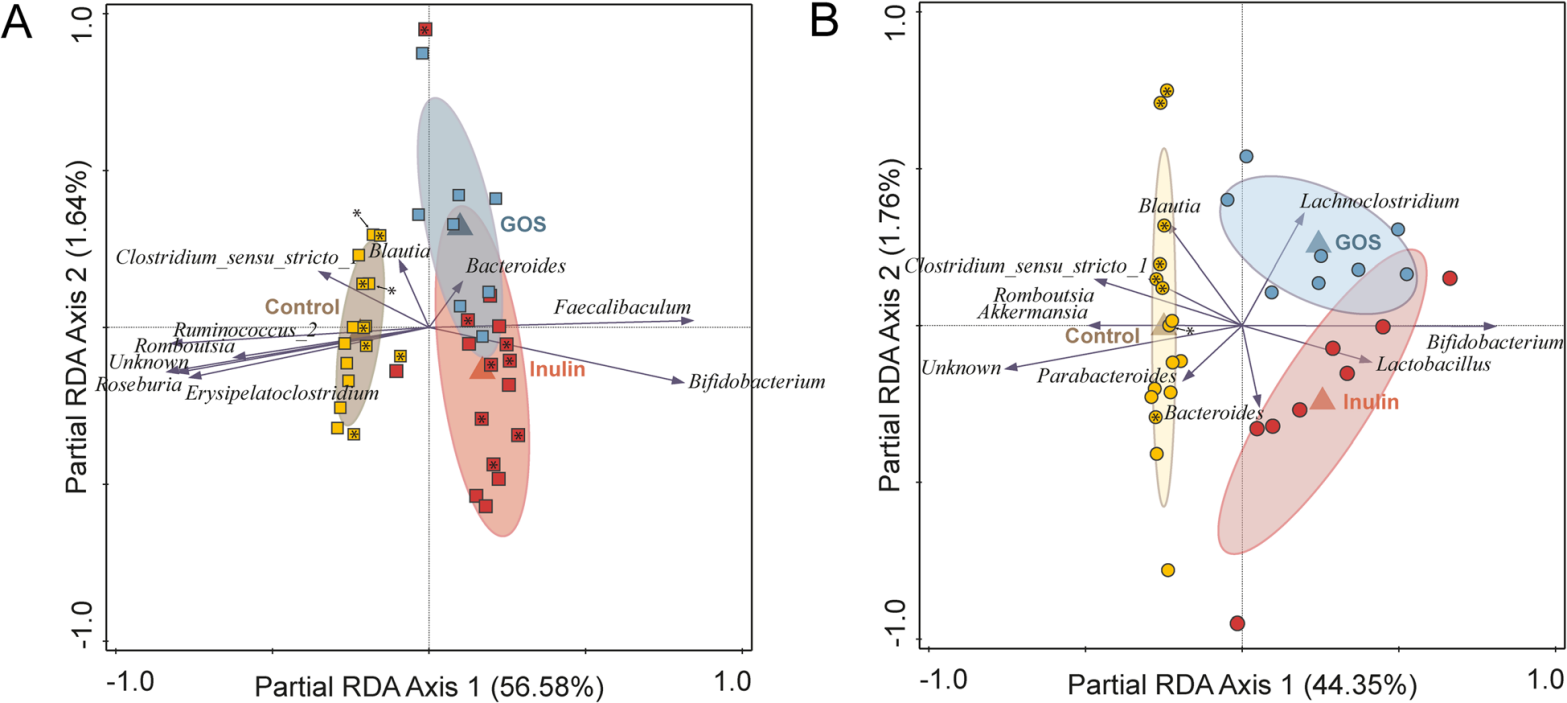
Effect of prebiotic supplementation depending on the dietary calcium phosphate background. **A** Partial redundancy analysis (pRDA) for the explanatory variable of supplementation (i.e., GOS, inulin, or control) (corrected for experiment) in the high-CaP_i_ diets: Hca (yellow squares), HcaGOS (blue squares), HcaInu (red squares). **B** Partial redundancy analysis (pRDA) for the explanatory variable of supplementation (i.e., GOS, inulin, or control) (corrected for experiment) in the low-CaP_i_ diets: Lca (yellow circles), LcaGOS (blue circles), LcaInu (red circles). Sample labels for experiment 1 and experiment 2 (Fig. 1F) contain an asterisk (*) or are empty, respectively

To further investigate the effects of supplementation (i.e., GOS or inulin), we performed multivariate analyses of the un-supplemented and supplemented variants of the diets separately for the high and low-CaP_i_ diets. In both the high and low-CaP_i_ dietary background, supplementation with GOS or inulin had a very large and significant impact on the microbiota composition, explaining 57% and 44% of the variation in high and low-CaP_i_ diets, respectively (Fig. 4A‑B). This analysis also revealed that only a modest separation between the two distinct supplements could be detected in either of the CaP_i_ diet backgrounds (explaining less than 2% of the overall variation), which is also seen in the un-supplemented diet groups particularly for the low-CaP_i_ diet. These observations indicate that GOS or inulin supplementation of the diet elicits very large but predominantly overlapping changes in the rat microbiome.

Irrespective of the CaP_i_ level in the diet, GOS or inulin supplementation resulted in a significantly increased relative abundance of the genus *Bifidobacterium* (ranging from 15 to 35%), although the effect size appeared to be larger in the low-CaP_i_ diet background (Fig. 3E). In the high-CaP_i_ diet background, the *Bifidobacterium* increase coincides with a parallel increase in relative abundance of the genus *Faecalibaculum*, which is not observed at all in the low-CaP_i_ diet background (Fig. 3F). Notably, the increased abundance of the family Erysipelotrichaecea was virtually exclusively attributable to the increased abundance of the *Faecalibaculum* genus, whereas other members (e.g., *Erysipelatoclostridium*) of this family remained unaffected (Figs. 1B and SF3). An increase in relative abundances of the *Lactobacillus* genus was detected upon the GOS and inulin supplementation of the low-CaP_i_ diet; however, this was only significant for GOS (*p* value of 0.02) and not for inulin (*p* value of 0.35) where the increase was driven by a few rats with very high abundance levels of this genus (10 to 30%). In addition, the relative abundance of *Bacteroides* increased in rats fed the inulin supplemented low-CaP_i_ diet relative to its GOS supplemented counterpart, although no inulin-supplementation associated increase in Bacteroides was detectable when comparing to the un-supplemented control diet. Notably, the latter observation could in part be driven by differences detected in the two independent experiments that were part of in this study (see above and Fig. 1F). The expansion of *Bifidobacterium* (and *Faecalibaculum* in the Hca diets) upon GOS or inulin supplementation, goes at the expense of the relative abundances of *Ruminoclostridium_9* (Fig. 3A) and the genera *Erysipelatoclostridum*, *Roseburia*, *Ruminococcus_2*, and *Romboutsia* (Figure SF4) in both the high- and low-CaP_i_ diet groups, despite the significant association of these genera with the un-supplemented high-CaP_i_ diet. In addition to these genera, also, the relative abundance of *Akkermansia* is reduced upon GOS or inulin supplementation to the low-CaP_i_ diet, although the populations remain relatively high (approximately twofold reduction in relative abundance) (Fig. 3B). Similarly, the *Blautia* populations decreased upon GOS or inulin supplementation in low-CaP_i_ diets, although these changes were not significant (Figure SF4). Finally, a decrease in *Parabacteroides* is seen in the low-CaP_i_ group but seems an effect exclusive to experiment 2 (Figure SF4). Remarkably, our analyses establish that the genera that are the strongest discriminants in the microbiota comparison between high- and low-CaP_i_ diets without supplementation are also the genera that are most strongly impacted by the addition of the GOS and inulin prebiotic supplements to these diets, which is apparently largely independent of the CaP_i_ level of the diet. Additionally, the analyses underpin that the microbiota changes elicited by these prebiotic supplements largely overlap and are more or less indistinguishable when comparing the two prebiotic supplementations in the context of diets with the same dietary CaP_i_ level. The latter conclusion is also supported by the absence of a significant link between microbiome at genus levels and supplement (*p* = 0.3). Thereby, these results establish that it does not matter whether GOS or inulin is used to supplement the diet in these experiments, but there is a significant and important prebiotic dependent co-modulation by the dietary CaP_i_ level of the diet.

### Prebiotic supplementation affects SCFA profiles in a calcium phosphate-dependent matter

**Fig. 5:**
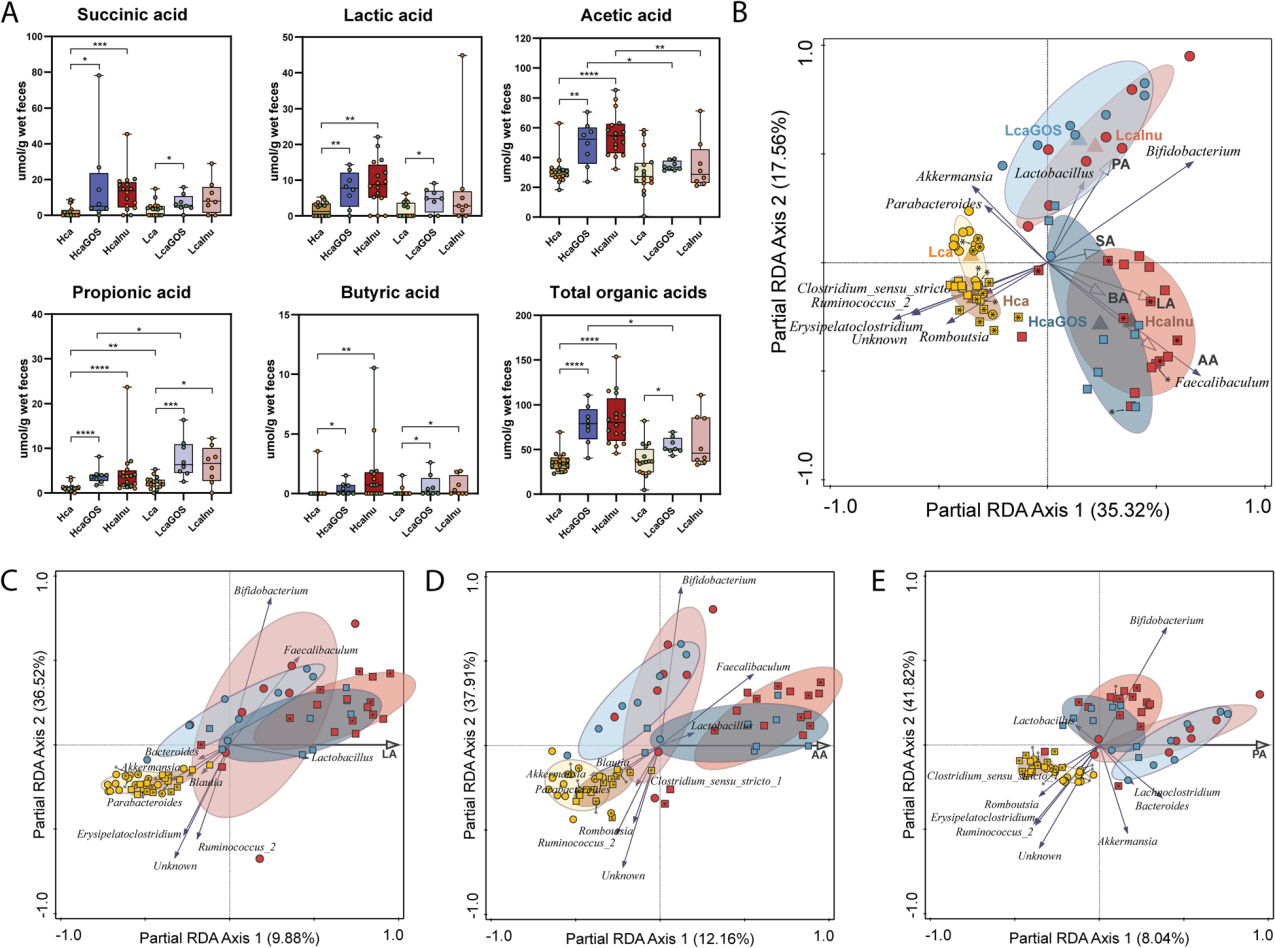
Organic acid profiles in fecal samples. **A** Succinic, lactic, acetic, propionic, butyric, and total organic acid levels of fecal samples expressed in μmol/g feces (wet weight). Asterisks (*) indicate significance after a two-sided Mann-Whitney *t* test. *p < 0.05; **p < 0.01; ***p < 0.001; ****p < 0.0001. Samples derived from experiment 1 and experiment 2 (Fig. 1F) are colored orange and green, respectively. **B** Partial redundancy analysis (pRDA) of microbiota at genus level with diet groups as explanatory variables with Hca (yellow squares), HcaGOS (blue squares), HcaInu (red squares), Lca (yellow circles), LcaGOS (blue circles), and LcaInu (red circles) and volatile acid profiles as supplementary variables with succinic acid (SA), lactic acid (LA), acetic acid (AA), propionic acid (PA), and butyric acid (BA). Partial redundancy analysis (pRDA) of microbiota at genus level with organic fecal acid concentrations as explanatory variable (corrected for experiment) showing the 10 most explanatory genera on the *x*-axis with **C** lactic acid (LA), **D** acetic acid (AA), and **E** propionic acid (PA). Sample labels for experiment 1 and experiment 2 (Fig. 1F) contain an asterisk (*) or are empty, respectively

The impact on the microbiota of the dietary variations (prebiotic and/or CaP_i_ level) may be reflected by bacterial metabolite profiles, like the carbohydrate fermentation metabolites lactic, succinic, acetic, propionic, and butyric acid. Therefore, the fermentation product profiles were determined in the fecal samples for which also 16S rRNA data was analyzed (Table ST1). Comparing the control diets that were not supplemented with prebiotics (i.e., Hca and Lca diets), propionic acid was significantly elevated in the Lca diet compared to the Hca diet, while all other organic acid levels were detected at comparable levels (Fig. 5A). Supplementation with GOS and inulin in the high-CaP_i_ diets, led to significant increases in all organic acid levels, with the strongest effect in the inulin supplemented diet (HcaInu) (Fig. 5A). These relatively consistent effects of prebiotic supplementation observed in the Hca diets are contrasted by the very modest effects of the same supplementations in the Lca diet. Propionic acid levels were predominantly increased in the LcaGOS and LcaInu diets, although modest increases were also seen in butyric acid and lactic acid, of which the latter was only significant in the LcaGOS diet (Fig. 5A). This CaP_i_-dependent contrast is also clearly reflected in the total amount of organic acids that are highly significantly increased in HcaGOS and HcaInu diets compared to the Hca diet, while in the low-CaP_i_ context only, the LcaGOS displayed a modest increase in total fecal organic acid levels (Fig. 5A).

pRDA with composition as response variable, diet as explanatory variable and organic acid profiles as supplementary variable reveals a correlation of propionic acid with the LcaGOS and LcaInu samples which are primarily associated with *Akkermansia*, *Bifidobacterium*, and *Lactobacillus* (Fig. 5B). Conversely, acetic and lactic acid are most abundant in the HcaGOS and HcaInu groups, associated with *Faecalibaculum.* The increase in succinic acid in the HcaGOS and HcaInu groups showed the smallest link to microbiome composition. Separate pRDAs, with individual acid levels as explanatory variable, indicate association of *Lactobacillus*, *Faecalibaculum*, and *Bifidobacterium* with increased lactic and acetic acid levels (Fig. 5C‑D). Furthermore, propionic acid levels are increased in samples with elevated *Bacteroides*, *Lachnoclostridium*, *Akkermansia*, and *Bifidobacterium* (Fig. 5E)*.*

## Discussion

In this study, we investigated the effect of dietary CaP_i_ and prebiotic supplementation (i.e., GOS or inulin) on the modulation of the endogenous microbiota in rats. Our study shows that the CaP_i_ background strongly influences gut microbiota composition, where diets high in CaP_i_ favor the proliferation of several Firmicutes members (among others *Romboutsia*, *Roseburia*, *Ruminoclostridium*, and *Ruminococcus*), while diets low in CaP_i_ predominantly favor *Bacteroides* and *Akkermansia*. Rats that were fed the un-supplemented high-CaP_i_ diet also had a higher relative abundance of *Lactobacillus* relative to those on un-supplemented low-CaP_i_ diets, which is in agreement with previous findings [14, 28]. The proposed proliferative effect of dietary CaP_i_ on lactobacilli and bifidobacteria [28, 37, 38] likely extends to other Gram-positive gut commensals as is seen in the increase of various genera belonging to the Firmicutes phylum in the high-CaP_i_ control diet, which contrasts with the low-CaP_i_ diets that favors the Gram-negative *Bacteroides* and *Akkermansia*. These observations agree with the established role of CaP_i_ in precipitation of secondary bile acids in the colon [37–39], creating physical-chemical conditions that are favoring the proliferation of Gram-positive bacteria [40]. Additionally, elevated levels of CaP_i_ could contribute to the buffering capacity of the colon lumen, which may also affect the microbiota. These mechanisms of action of dietary CaP_i_ are very likely to be conserved in humans, although there are to the best of our knowledge no reports dedicated to the investigation of the influence of dietary CaP_i_ intake on human gut-microbiota composition.

The significant low-CaP_i_-related increase in intestinal *Akkermansia* is noteworthy, as this genus is a dedicated mucin degrader that can also stimulate mucus production, and is associated with improved insulin resistance and improved mucosal barrier function [54–56]. However, other studies have shown that a disbalance in *Akkermansia* populations can negatively affect mucosal barrier function, as is illustrated by the elevated *Akkermansia* levels associated with dietary heme supplementation in mice that was associated with disproportionate mucolysis and colonic epithelial hyperproliferation and reduced mucosal barrier function [35]. Similarly, gnotobiotic mice colonized with a synthetic commensal microbiota cocktail, containing among others *Akkermansia muciniphila* and several *Bacteroides* strains was shown to switch to degradation of the colonic mucus layer when deprived of dietary fiber, resulting in an increased susceptibility to pathogens that can traverse the mucosal barrier [57]. Interestingly, the same study also shows that diet-supplementation with purified soluble fibers, including inulin (as well as arabinoxylan and β-glucan), could barely mitigate the observed microbial erosion of the mucus layer, suggesting that complex plant-derived fibers are critical ingredients in the diet. Interestingly, *Bacteroides thetaiotaomicron*, when mono-associated in mice, metabolized predominantly the host mucus glycans only when the mice were fed a diet that lacked non-digestible plant-derived polysaccharides [58], underpinning the role of dietary fibers in modulation of not only the endogenous microbiota composition and activity but also its interaction with the host. Since the mucin-degrading bacteria *Akkermansia* and *Bacteroides* are enriched in our experimental rats fed a low-CaP_i_ diet, it is tempting to speculate that the mucus layer is also thinner in these animals as compared to their high-CaP_i_ diet counterparts, which is potentially compensated (in part) by prebiotic supplementation (i.e., GOS or inulin). The elevated levels of these bacterial groups in the low-CaP_i_-diet context are in good agreement with the significantly elevated levels of fecal propionic acid, which is a known fermentation product of these bacteria [59–61]. Since prebiotic supplementation (either GOS or inulin) in a low-CaP_i_-diet context led to a further elevation of propionic acid levels without strongly affecting the other organic acids, it is likely that these same bacterial groups are also involved in metabolization of these supplements. Therefore, our results suggest that CaP_i_ could be a determinant for the consequences of prebiotic supplementation on the modulation of the microbiota and the organic acid profiles. Furthermore, such consequences could have an accessory impact on the host mucus layer. Analogous to what we concluded above, these findings clearly warrant human studies investigating the role of dietary CaP_i_ in modulating the gut microbiota composition in combination with its effects on mucosal barrier function.

Supplementation with GOS and inulin-elicited congruent effects within each CaP_i_ background. Both CaP_i_ backgrounds facilitated large increases in local *Bifidobacterium* abundance which has been commonly observed for inulin and fructo-oligosaccharides (FOS) [13–15, 19, 62, 63] and GOS [10, 11]. Importantly, in our study, the increase of the *Bifidobacterium* genus in both the GOS and inulin supplemented diets is not attributable to different *Bifidobacterium* ASV’s for the two prebiotic substrates (data not shown), further supporting the congruent effects of GOS and inulin. Interestingly, only the high-CaP_i_ background substantially elevated *Faecalibaculum* populations, suggesting that the Gram-positive *Faecalibaculum* may be particularly susceptible to (secondary) bile acid associated stress. GOS and inulin differ substantially in chemical structure and complexity [4, 5] and consequently also the mechanism of their degradation and utilization by members of the gut microbiome. Fructo-oligosaccharides (FOS) and inulin are regularly hydrolyzed extracellularly by bifidobacteria but the capacity to degrade FOS and inulin of different lengths is strain-dependent [64]. GOS utilization is diverse in *Bifidobacterium* and a multitude of extra- and intracellular hydrolases and import systems have been attributed to different species and have also shown to be strain specific [6, 65–67]. Lactobacilli have limited capacity to utilize GOS or inulin and commonly rely on intracellular GOS hydrolysis [68–70], and extracellular digestion of inulin has been described for several species [71–73]. GOS metabolism is often facilitated by ABC transporters and occasionally by lactose permeases in *Bifidobacterium* species [65, 66, 74], while several *Lactobacillus* species (i.e., *L. acidophilus*, *L. reuteri* and *L. plantarum)* employ lactose permeases for GOS import [68–70]. To the best of our knowledge, the mechanisms of GOS or inulin utilization in *Faecalibaculum* has not been described to date. Public genomes of *Faecalibaculum rodentium* encode secreted proteins with glycoside hydrolase (GH) family 32 domains that are commonly involved in inulin utilization [75]. There are no indications of extracellular GOS degradation in *Faecalibaculum.* However, *Faecalibaculum rodentium* genomes do encode lactose-PTS systems as well as genetically linked intracellular 6-P-β-galactosidases, which are functions that in *Lactobacillus gasseri* were shown to convey the ability to utilize GOS [76, 77]. Therefore, it is conceivable that *Faecalibaculum* can also utilize GOS through PTS-driven import. Moreover, since the bulk of the GOS constituents used in this study have a degree of polymerization of 3 to 4, they are likely not dependent on extracellular degradation, and thereby could readily stimulate *Faecalibaculum* growth. Additionally, due to declining lactase activity in maturing rats [78], lactose is poorly broken down and absorbed in the small intestine and may still stimulate the endogenous microbiota, including *Faecalibaculum*. Overall, it is clear that *Faecalibaculum* profits from the high-CaP_i_ environment and is equipped to compete against endogenous Bifidobacteria in both prebiotic supplemented diets (i.e., HcaGOS and HcaInu diets). Intriguingly, it has been postulated that the obligate anaerobic *Faecalibaculum rodentium* can out-compete endogenous *Lactobacillus* and *Bifidobacterium* populations during gut maturation in rodents as the gut becomes more strictly anaerobic [79]. Notably, the murine *Faecalibaculum rodentium* and its human counterpart, *Holdemanella biformis*, were recently associated with anti-tumorigenic activity [80], potentiating their positive health benefits on both the murine and human host, respectively. Moreover, we have recently shown that the Hca diet favors the intestinal persistence of dietary lactobacilli (e.g., probiotics) compared to the Lca diet [73], further supporting the health-beneficial potential of high-CaP_i_ diets. Contrary to the dietary lactobacilli, the endogenous intestinal lactobacilli were particularly enriched upon prebiotic supplementation in the low-CaP_i_ diet background [73]. This result appears to be in contrast to previous reports about significantly increased *Lactobacillus* populations upon inulin supplementation in diets with either low- or high-CaP_i_ levels [14]. Although the latter study used virtually identical CaP_i_ regimes as we employed in the present study, the level of inulin supplementation was significantly higher (60 g/kg compared to 40 g/kg in the current study). It is possible that in our experiments the lower level of prebiotic-supplementation diminished the *Lactobacillus* stimulation effect observed earlier, possibly due to more stringent prebiotic substrate competition (i.e., in HcaGOS and HcaInu diets) by endogenous *Bifidobacterium* and *Faecalibaculum* populations.

*Bifidobacterium*, *Faecalibaculum*, and *Lactobacillus* are all lactic acid producers [79, 81, 82], but they can also produce acetic acid to a variable degree [79, 82–84]. The effect of GOS and inulin supplementation on organic acid profiles is very much CaP_i_ dependent and increases in fecal lactic and acetic acid levels are especially associated with the supplemented high-CaP_i_ diets. This is remarkable because the Bifidobacteria are greatly increased in diets supplemented with GOS or inulin irrespective of the CaP_i_ level of the diet, while their signature metabolites (i.e., lactic and acetic acid) were predominately increased in the supplemented high-CaP_i_ diets, although modestly increased levels of lactic acid were also detected in the low CaP_i_, GOS supplemented diet. Fecal measurements of organic acid profiles are straightforward and convenient and often determined in diet-intervention studies that employ carbohydrate supplementations. However, the dynamic process of organic acid nutrikinetics (i.e., the overall resultant of microbial production, host absorption, and bacterial cross-feeding) complicate their accurate interpretation in the context of dietary intervention and microbial composition and/or activity [85]. For example, another study that investigated the interplay between GOS and dietary CaP_i_ collected proximal colonic contents instead of feces and concluded that high-CaP_i_ regimes counteracted lactate and SCFA production [86], while our measurements from feces show the contrary. Nonetheless, the effect of dietary CaP_i_ and prebiotic supplementation clearly influences the fecal organic acid levels, and the detected correlation of increased lactic and acetic acid with bacterial genera that produce these acids in high-CaP_i_ diets is intriguing and suggests that these effects may also lead to a more acidified gut lumen, which could further influence the intestinal microbiota and its interaction with the host.

It is important to note that the interaction of CaP_i_ and other (micro)nutrients is not restricted to prebiotics. Particularly, there are several reports on the role dietary CaP_i_ can play in the suppression of colonic cytotoxicity levels that are associated with the consumption of heme (i.e., red meat) and that were shown to induce colonic epithelial hyperproliferation [41, 42, 87]. Human relevance of this activity of dietary CaP_i_ is apparent from epidemiological evidence that establishes the association between the consumption of heme and an increased risk for the development of colon cancer [88, 89]. In the context of heme containing diets, high-CaP_i_ levels comparable to the levels employed in this study, prevented the heme-induced cytolytic activity of fecal water in mice [41]. Strikingly, dietary heme also affects the microbiota leading to decreased Firmicutes and increased Bacteroidetes in the colon of mice, which was proposed to be related to the selective susceptibility of Gram-positive bacteria to the heme-induced cytotoxic fecal water [90]. Moreover, the heme-induced hyperproliferation was recently demonstrated to be dependent on the microbiota, identifying an association of the elevated levels of *Akkermansia municiphila* in the intestine of mice that were fed a heme-containing diet with disproportionate mucolysis and reduced mucosal barrier function, thereby potentiating colonic epithelial hyperproliferation and enhancing colon cancer risk [35]. The proposed role of CaP_i_ in reducing the heme-induced cytotoxicity in the colon displays strong similarity with its precipitating effect on colonic secondary bile acids, which also suppresses the mucosa-damaging physical-chemical characteristics of the colon content, indicating a consistent mode of action of dietary CaP_i_ in reducing colon cytotoxicity through the complexation of damaging components from the lumen content. These effects were mostly evidenced in rodent models, but probably are conserved in humans and other monogastric animals based on the conservation of the physical chemical processes involved. This notion is supported by the observations in human studies that have shown that high-CaP_i_ diets are protective against ETEC-induced diarrhea [29] and are able to counteract the impairment of mucosal barrier function in individuals consuming a diet supplemented with high levels of FOS [15].

Taken together, dietary CaP_i_ can significantly affect physical chemical conditions in the colon lumen, and in human studies was evidenced to protect against infection and barrier impairment associated with prebiotic consumption, as well as the reduction of colon cancer risk, particularly in association with the consumption of red meat (i.e., heme). The interaction of nutrients that can have potential negative health effects by their capacity to impair colonic barrier function (i.e., heme and FOS) with CaP_i_ appears to be the common theme in these findings, where there is increasing evidence for a pivotal role of the microbiota in both the negative effects of some of these nutrients (i.e., heme) as well as the protective effect of supplementation with high levels of CaP_i_. This study exemplifies the important impact of dietary CaP_i_ on the microbiota in rats and highlights the interaction of this micro-nutrient with prebiotic supplements in co-modulation of the intestinal microbiota, underpinning the need for similar human studies that also assess important gut-health parameters like fecal water cytotoxicity and colon barrier function.

## Conclusions

Our study exemplifies that GOS or inulin supplementation has a profound and congruent effect on the endogenous microbiota and its fermentation output, which is strongly dependent on the dietary CaP_i_ level. It is likely that the endogenous *Bifidobacterium* and *Faecalibaculum* members of the microbiota are the most effective utilizers of both these substrates despite the differences in their chemical structure and complexity. Nevertheless, the modulatory capacity of other candidate prebiotics may modulate the microbiota in a different manner, depending on the genus- and species-specific enzymatic repertoires of the endogenous microbiota members. The diet-related microbiota responsiveness is also strongly determined by the genera that are associated with the un-supplemented Hca and Lca diets (e.g., *Ruminoccocus*, *Romboutsia*, *Ruminoclostridium*, and *Akkermansia*), which appear to decrease in relative abundance to accommodate the prebiotic-induced proliferation of *Bifidobacterium* and *Faecalibaculum*. Our study concludes unequivocally that the gut ecosystem responsiveness is determined not only by specific nutrients or supplements (i.e., dietary fiber or prebiotics) but is also highly dependent on secondary micronutrients such as CaP_i_. In many cases, micronutrients can steer the dynamics of microbial gut populations in conjunction with the selective forces exerted by macronutrients, potentially stimulating commensals and pathogens in similar and/or distinct manners, which can lead to shifts in the microbial ecosystem balance that can have consequences for health. Our results underpin that further investigation of the interplay between macro- (fibers or prebiotics) and micronutrients is required to better predict the microbiome and physico-chemical responses to dietary interventions with functional ingredients like prebiotics.

## Supplementary Information

**Additional file 1: Figure SF1.** Experimental set up of animal trials. Two independent animal trials with A) Hca, HcaInu, Lca and LcaInu diets and B) Hca, HcaGOS, HcaInu, Lca and LcaGOS diets. All rats were acclimatized to the experimental diets fourteen days. Feed intake was monitored daily while animals were weighed twice per week. After the fourteen-day acclimatization period, fecal samples were collected for each rat. **Figure SF2.** Individual composition plots. Individual stacked bar plots of relative abundance of A) Phylum level and B) Family level. **Figure SF3.** Rat weight over time. Weight of individual rats and averages with SEM for each dietary group from day of arrival (day 0) to the day of fecal pellet collection (day 14) for A) Experiment 1, with rats on the Lca diet (grey circles), LcaInu diet (grey triangles), Hca diet (black circles), and HcaInu (black triangles), and B) Experiment 2, with rats on the Lca diet (grey circles), LcaGOS diet (grey squares), Hca diet (black circles), HcaGOS diet (black squares) and HcaInu diet (black triangles). Boxplots of average and individual relative weight increase of each rat from day 14 compared to day 0 for C) Experiment 1, and D) Experiment 2. Differences in relative weight increase for each group were not significant. **Figure SF4.** Univariate analysis of discriminant genera between dietary groups. Relative abundance of the genera *Romboutsia, Roseburia, Blautia, Ruminococcus_2, Erysipelatoclostridium* and *Parabacteroides* in each dietary group. Asterisks (*) indicate significance after a two-sided Mann-Whitney t-test. *: *p* < 0.05; **p: < 0.01; ***: p < 0.001; ****: *p* < 0.000. Samples derived from experiment 1 and experiment 2 (Fig. 1F) are colored orange and green, respectively. **Supplemental Table ST1.** Rat metadata and fecal organic acid levels. Rat metadata used in both studies, including sample alias and accession numbers as deposited in ENA, study numbers corresponding to ENA submissions, experiment numbers corresponding to the current manuscript, rat numbers, dietary supplementation and fecal acid concentrations. CaPi: Calcium phosphate; Hca: high CaPi; Lca: low CaPi; Inu: inulin; GOS: galacto-oligosaccharides; Cl: cellulose; SA: succinic acid; LA: lactic acid; AA: acetic acid; PA: propionic acid; BA: butyric acid.

## Data Availability

The 16S ribosomal RNA data used in this study was deposited at the European Nucleotide Archive (ENA) with the following accession numbers: ERS5847309 to ERS5847340 and ERS5847673 to ERS5847712, under study number PRJEB43382. A corresponding metadata sheet (Table ST1) is found in the supplementary materials.
