# Supplementary material for: Dietary calcium phosphate strongly impacts gut microbiome changes elicited by inulin and galacto-oligosaccharides consumption
Source: Microbiome. 2021 Nov 4;9:218. doi: 10.1186/s40168-021-01148-0 (PMC8567720; doi:10.1186/s40168-021-01148-0)
Supplement: Supplementary file 2 — Additional file 1: Figure SF1. Experimental set up of animal trials. Two independent animal trials with A) Hca, HcaInu, Lca and LcaInu diets and B) Hca, HcaGOS, HcaInu, Lca and LcaGOS diets. All rats were acclimatized to the experimental diets fourteen days. Feed intake was monitored daily while animals were weighed twice per week. After the fourteen-day acclimatization period, fecal samples were collected for each rat. Figure SF2. Individual composition plots. Individual stacked bar plots of relative abundance of A) Phylum level and B) Family level. Figure SF3. Rat weight over time. Weight of individual rats and averages with SEM for each dietary group from day of arrival (day 0) to the day of fecal pellet collection (day 14) for A) Experiment 1, with rats on the Lca diet (grey circles), LcaInu diet (grey triangles), Hca diet (black circles), and HcaInu (black triangles), and B) Experiment 2, with rats on the Lca diet (grey circles), LcaGOS diet (grey squares), Hca diet (black circles), HcaGOS diet (black squares) and HcaInu diet (black triangles). Boxplots of average and individual relative weight increase of each rat from day 14 compared to day 0 for C) Experiment 1, and D) Experiment 2. Differences in relative weight increase for each group were not significant. Figure SF4. Univariate analysis of discriminant genera between dietary groups. Relative abundance of the genera Romboutsia, Roseburia, Blautia, Ruminococcus_2, Erysipelatoclostridium and Parabacteroides in each dietary group. Asterisks (*) indicate significance after a two-sided Mann-Whitney t-test. *: p < 0.05; **p: < 0.01; ***: p < 0.001; ****: p < 0.000. Samples derived from experiment 1 and experiment 2 (Fig. 1F) are colored orange and green, respectively. Supplemental Table ST1. Rat metadata and fecal organic acid levels. Rat metadata used in both studies, including sample alias and accession numbers as deposited in ENA, study numbers corresponding to ENA submissions, experiment numbers correspondin [file 40168_2021_1148_MOESM2_ESM.pdf]

Supplementary Materials

Dietary calcium phosphate strongly impacts gut microbiome changes elicited by inulin and galacto-oligosaccharides consumption

J. Fuhren[1], M. Schwalbe[1], J. Boekhorst[1], C. Rösch[2], H.A. Schols[2], M. Kleerebezem[1]

1: Host Microbe Interactomics Group, Wageningen University & Research, Wageningen, the Netherlands

2: Laboratory of Food Chemistry, Wageningen University & Research, Wageningen, the Netherlands

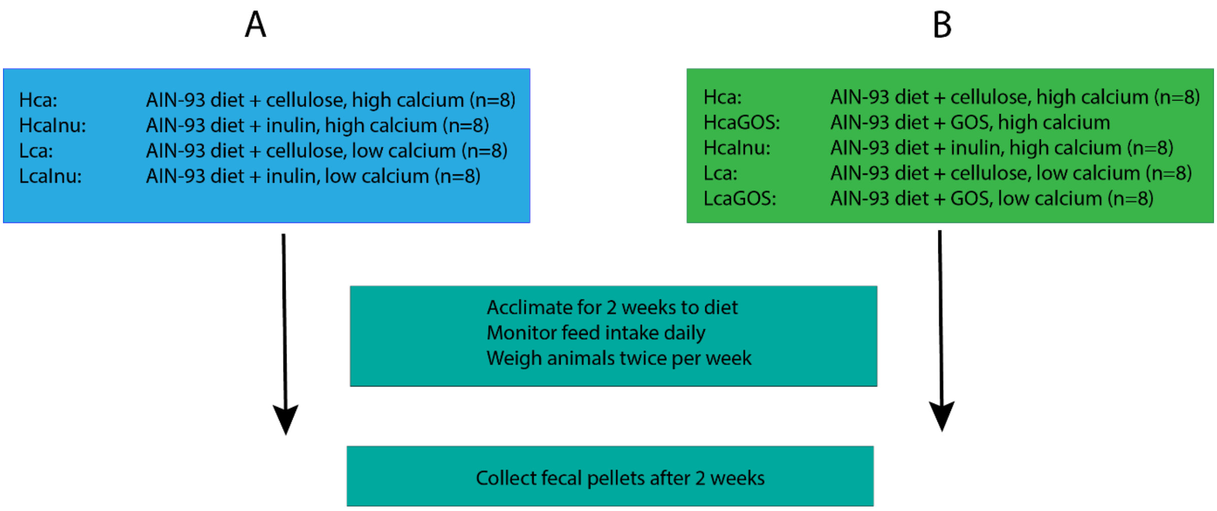

Figure SF1. Experimental set up of animal trials. Two independent animal trials with A) Hca, HcaInu, Lca and LcaInu diets and B) Hca, HcaGOS, HcaInu, Lca and LcaGOS diets. All rats were acclimatized to the experimental diets fourteen days. Feed intake was monitored daily while animals were weighed twice per week. After the fourteen-day acclimatization period, fecal samples were collected for each rat.

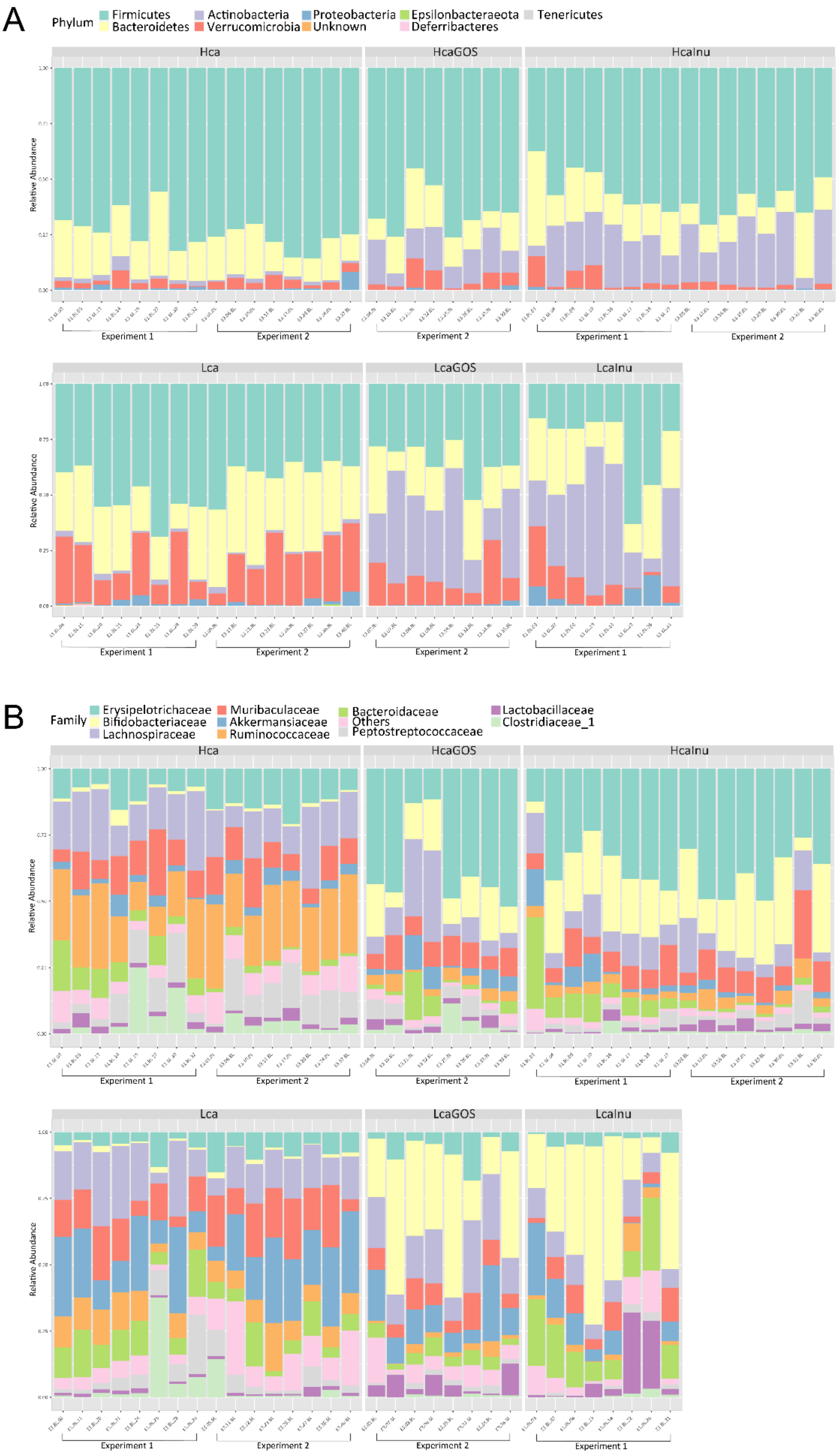

Figure SF2. Individual composition plots. Individual stacked bar plots of relative abundance of A) Phylum level and B) Family level.

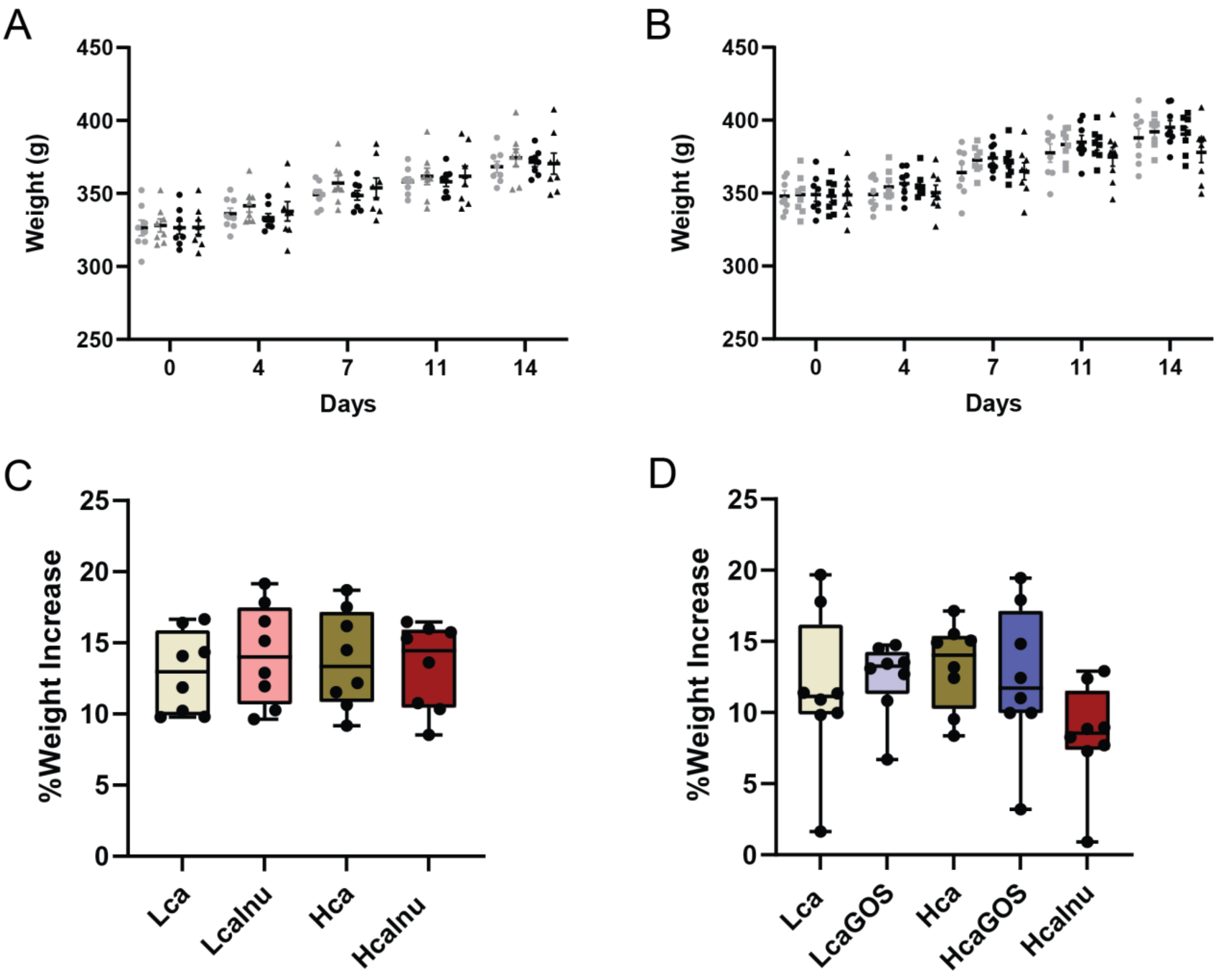

Figure SF3. Rat weight over time. Weight of individual rats and averages with SEM for each dietary group from day of arrival (day 0) to the day of fecal pellet collection (day 14) for A) Experiment 1, with rats on the Lca diet (grey circles), LcaInu diet (grey triangles), Hca diet (black circles), and HcaInu (black triangles), and B) Experiment 2, with rats on the Lca diet (grey circles), LcaGOS diet (grey squares), Hca diet (black circles), HcaGOS diet (black squares) and HcaInu diet (black triangles). Boxplots of average and individual relative weight increase of each rat from day 14 compared to day 0 for C) Experiment 1, and D) Experiment 2. Differences in relative weight increase for each group were not significant.

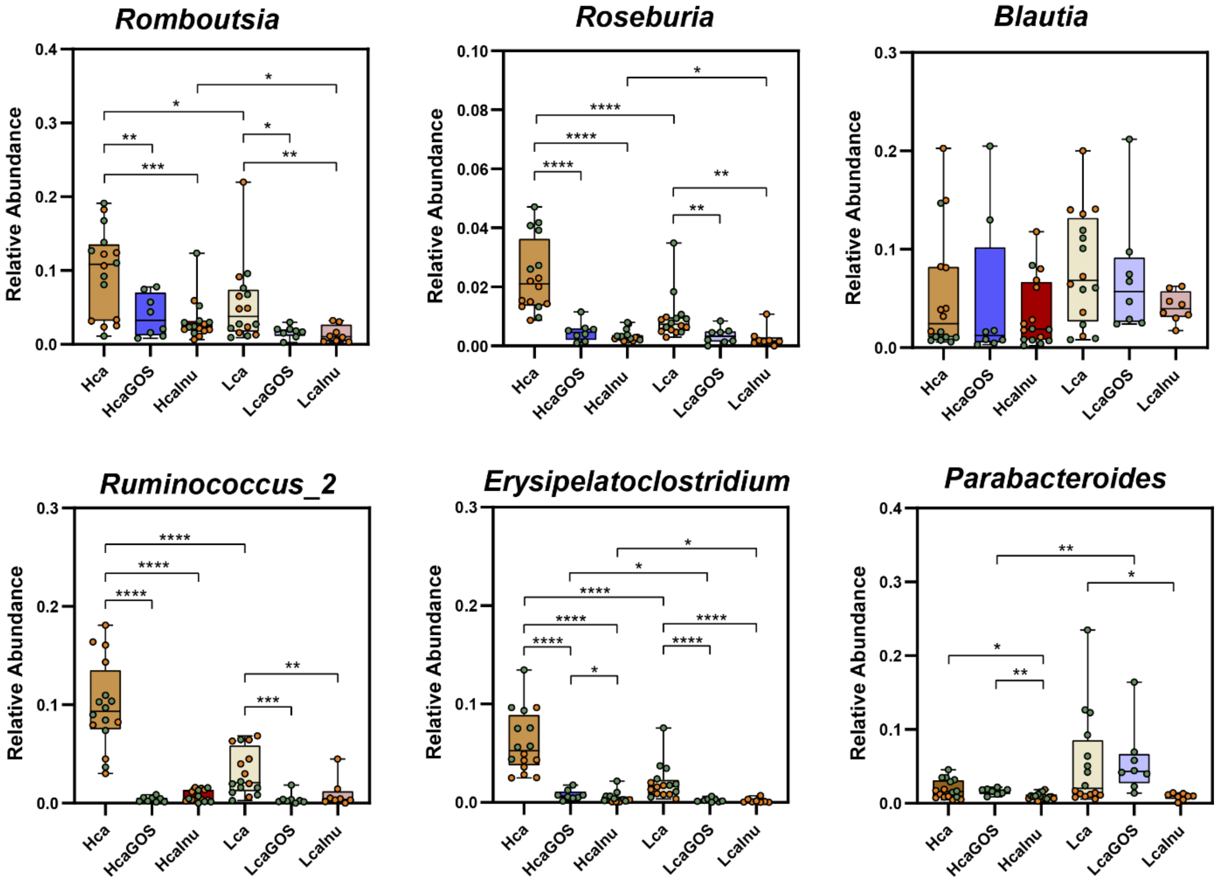

Figure SF4. Univariate analysis of discriminant genera between dietary groups. Relative abundance of the genera *Romboutsia, Roseburia, Blautia, Ruminococcus_2, Erysipelatoclostridium* and *Parabacteroides* in each dietary group. Asterisks (*) indicate significance after a two-sided Mann-Whitney t-test. *: $p < 0.05$; **p: < 0.01; ***: $p < 0.001$; ****: $p < 0.000$. Samples derived from experiment 1 and experiment 2 (Figure 1F) are colored orange and green, respectively

Supplemental Table ST1: Rata metadata and fecal organic acid levels. Rat metadata used in both studies, including sample alias and accession numbers as deposited in ENA, study numbers corresponding to ENA submissions, experiment numbers corresponding to the current manuscript, rat numbers, dietary supplementation and fecal acid concentrations. CaP_i: Calcium phosphate; Hca: high CaP_i; Lca: low CaP_i; Inu: inulin; GOS: galacto-oligosaccharides; Cl: cellulose; SA: succinic acid; LA: lactic acid; AA: acetic acid; PA: propionic acid; BA: butyric acid.

Rat Metadata							Fecal organic acid concentrations (umol/g wet weight feces)				
**Sample alias (ENA)**	**Primary Accession (ENA)**	**Study # (ENA)**	**Exp #**	**Rat #**	**CaPi**	**Supplement**	**SA**	**LA**	**AA**	**PA**	**BA**
1	ERS5847309	1	1	1	Hca	Inu	45.482	0.000	79.110	23.649	5.322
2	ERS5847310	1	1	2	Hca	Cl	0.000	3.236	26.874	3.462	3.562
3	ERS5847311	1	1	3	Lca	Inu	8.371	0.000	21.117	3.612	0.000
4	ERS5847312	1	1	4	Hca	Inu	0.000	5.020	46.425	2.092	0.000
5	ERS5847313	1	1	5	Hca	Cl	0.000	5.265	63.085	1.307	0.000
6	ERS5847314	1	1	6	Lca	Cl	0.000	0.000	23.172	1.950	0.000
7	ERS5847315	1	1	7	Lca	Inu	0.000	5.016	22.560	8.044	0.292
8	ERS5847316	1	1	8	Lca	Inu	7.464	2.804	26.414	12.243	1.825
9	ERS5847317	1	1	9	Hca	Inu	19.042	0.000	32.202	5.207	0.000
10	ERS5847318	1	1	10	Hca	Inu	7.053	8.370	61.863	6.573	1.666
11	ERS5847319	1	1	11	Lca	Cl	0.000	0.000	35.143	2.510	0.000
12	ERS5847320	1	1	12	Hca	Cl	0.000	0.000	26.875	0.000	0.000
13	ERS5847321	1	1	13	Lca	Inu	0.000	1.622	31.086	5.631	0.000
14	ERS5847322	1	1	14	Hca	Cl	0.000	3.781	38.146	2.845	0.000

15	ERS5847323	1	1	15	Hca	Cl	0.000	0.000	24.961	0.628	0.000
16	ERS5847324	1	1	16	Hca	Inu	4.122	0.000	40.552	1.118	0.000
17	ERS5847325	1	1	17	Hca	Inu	12.739	9.605	41.798	3.744	0.000
18	ERS5847326	1	1	18	Lca	Inu	5.649	2.821	24.148	7.628	0.744
19	ERS5847327	1	1	19	Hca	Inu	19.598	15.046	74.791	4.591	1.234
20	ERS5847328	1	1	20	Lca	Cl	0.000	4.319	48.436	4.288	0.000
21	ERS5847329	1	1	21	Lca	Cl	0.000	0.000	22.778	1.416	0.000
22	ERS5847330	1	1	22	Lca	Inu	16.963	44.840	48.934	0.000	0.000
23	ERS5847331	1	1	23	Hca	Inu	5.245	9.203	41.553	1.025	0.000
24	ERS5847332	1	1	24	Lca	Cl	5.160	0.000	17.650	0.918	0.000
25	ERS5847333	1	1	25	Lca	Cl	5.180	0.000	25.934	2.536	0.489
26	ERS5847334	1	1	26	Lca	Inu	12.550	0.000	71.328	2.369	0.000
27	ERS5847335	1	1	27	Hca	Cl	7.583	0.000	33.176	1.296	0.000
28	ERS5847336	1	1	28	Lca	Cl	5.484	3.912	25.429	1.905	0.000
29	ERS5847337	1	1	29	Lca	Cl	14.843	6.167	58.308	2.973	0.000
30	ERS5847338	1	1	30	Hca	Cl	3.239	3.636	30.296	0.743	0.000
31	ERS5847339	1	1	31	Lca	Inu	28.974	7.531	35.982	10.753	1.895
32	ERS5847340	1	1	32	Hca	Cl	7.134	0.000	27.705	1.063	0.000
3.01	ERS5847673	3	2	1	Hca	Cl	0.000	0.000	33.026	1.174	0.000
3.02	ERS5847674	3	2	2	Lca	GOS	7.356	0.000	32.517	10.256	0.496
3.03	ERS5847675	3	2	3	Hca	Inu	14.703	7.908	53.037	1.754	0.000
3.04	ERS5847676	3	2	4	Hca	GOS	2.024	1.528	33.718	3.114	0.000
3.05	ERS5847677	3	2	5	Lca	Cl	3.240	3.839	24.362	2.646	1.533
3.06	ERS5847678	3	2	6	Hca	Cl	8.730	4.464	30.961	0.921	0.000
3.07	ERS5847679	3	2	7	Lca	GOS	5.188	9.155	39.244	4.383	0.000

3.08	ERS5847680	3	2	8	Lca	GOS	11.891	0.000	38.675	16.344	2.619
3.09	ERS5847681	3	2	9	Lca	GOS	4.234	6.431	33.198	4.840	0.000
3.10	ERS5847682	3	2	10	Hca	Cl	0.000	0.000	30.493	1.296	0.000
3.11	ERS5847683	3	2	11	Lca	Cl	0.000	0.000	30.832	5.275	0.000
3.12	ERS5847684	3	2	12	Lca	Cl	0.000	0.000	55.766	0.690	0.000
3.13	ERS5847685	3	2	13	Hca	Inu	20.259	15.628	63.038	3.680	10.531
3.14	ERS5847686	3	2	14	Hca	Cl	0.000	0.000	31.775	0.382	0.000
3.15	ERS5847687	3	2	15	Hca	GOS	14.333	12.846	49.577	3.569	1.519
3.16	ERS5847688	3	2	16	Hca	Inu	17.098	11.216	58.538	1.307	0.746
3.17	ERS5847689	3	2	17	Hca	Cl	1.682	2.525	18.375	0.499	0.000
3.18	ERS5847690	3	2	18	Hca	Inu	0.000	6.308	57.194	7.131	0.708
3.19	ERS5847691	3	2	19	Lca	GOS	6.015	7.224	31.448	5.835	0.391
3.20	ERS5847692	3	2	20	Hca	Cl	0.000	0.000	23.545	1.180	0.000
3.21	ERS5847693	3	2	21	Hca	GOS	78.088	0.000	23.727	8.143	0.685
3.22	ERS5847694	3	2	22	Hca	GOS	26.765	9.950	57.340	3.977	0.000
3.23	ERS5847695	3	2	23	Lca	Cl	3.435	2.823	28.498	1.929	0.000
3.24	ERS5847696	3	2	24	Hca	Cl	2.026	2.843	30.094	0.913	0.000
3.25	ERS5847697	3	2	25	Hca	GOS	3.947	14.359	55.200	1.757	0.751
3.26	ERS5847698	3	2	26	Lca	Cl	4.133	2.447	32.680	2.963	0.000
3.27	ERS5847699	3	2	27	Lca	Cl	9.949	3.159	36.871	3.535	0.000
3.28	ERS5847700	3	2	28	Hca	GOS	6.205	5.664	42.349	4.002	0.000
3.29	ERS5847701	3	2	29	Hca	Inu	16.924	19.564	50.231	1.560	1.837
3.30	ERS5847702	3	2	30	Hca	Inu	9.295	22.108	48.901	2.450	0.719
3.31	ERS5847703	3	2	31	Hca	Inu	3.226	5.243	56.269	3.870	0.000
3.32	ERS5847704	3	2	32	Lca	GOS	0.000	4.856	35.653	6.856	1.573

3.33	ERS5847705	3	2	33	Hca	GOS	0.000	7.324	61.161	2.528	0.423
3.34	ERS5847706	3	2	34	Lca	GOS	15.712	3.981	33.879	11.123	0.000
3.35	ERS5847707	3	2	35	Hca	Inu	14.811	12.086	85.387	4.105	1.892
3.36	ERS5847708	3	2	36	Lca	Cl	3.428	0.000	15.097	1.751	0.000
3.37	ERS5847709	3	2	37	Hca	Cl	0.000	2.941	28.170	1.013	0.000
3.38	ERS5847710	3	2	38	Lca	GOS	4.395	4.880	31.204	2.564	0.000
3.39	ERS5847711	3	2	39	Hca	GOS	3.798	8.205	70.679	4.185	0.000
3.40	ERS5847712	3	2	40	Lca	Cl	4.050	0.000	0.620	0.000	0.000
